# A data-driven approach to the semantics of iconicity in American Sign Language and English

**DOI:** 10.1017/langcog.2019.52

**Published:** 2020-03-02

**Authors:** BILL THOMPSON, MARCUS PERLMAN, GARY LUPYAN, ZED SEVCIKOVA SEHYR, KAREN EMMOREY

**Affiliations:** University of California, Berkeley; University of Birmingham; University of Wisconsin-Madison; San Diego State University; San Diego State University

**Keywords:** iconicity, computational semantics, American Sign Language, language modality, lexicon

## Abstract

A growing body of research shows that both signed and spoken languages display regular patterns of iconicity in their vocabularies. We compared iconicity in the lexicons of American Sign Language (ASL) and English by combining previously collected ratings of ASL signs (Caselli, Sevcikova Sehyr, Cohen-Goldberg, & Emmorey, 2017) and English words (Winter, Perlman, Perry, & Lupyan, 2017) with the use of data-driven semantic vectors derived from English. Our analyses show that models of spoken language lexical semantics drawn from large text corpora can be useful for predicting the iconicity of signs as well as words. Compared to English, ASL has a greater number of regions of semantic space with concentrations of highly iconic vocabulary. There was an overall negative relationship between semantic density and the iconicity of both English words and ASL signs. This negative relationship disappeared for highly iconic signs, suggesting that iconic forms may be more easily discriminable in ASL than in English. Our findings contribute to an increasingly detailed picture of how iconicity is distributed across different languages.

## 1. Introduction

With the increasing recognition that iconicity – broadly defined as the perceived resemblance between form and meaning – is a fundamental property of human language, researchers are now beginning to uncover the detailed, systematic patterns that characterize iconicity across specific languages, including signed and spoken languages (Dingemanse, Blasi, Lupyan, Christiansen, & Monaghan, 2015; Imai & Kita, 2014; Meir, 2010; Padden, Hwang, Lepic, & Seegers, 2015; Perlman, Little, Thompson, & Thompson, 2018; Perniss, Thompson, & Vigliocco, 2010). This work seeks to understand how iconicity is woven through the grammar, lexicon, phonology, gesture, and prosody of different languages, with particular attention to how these patterns reflect the functions of iconicity in language processing and learning.

In the current study, we examine where in a language’s vocabulary iconicity is most concentrated. In particular, we use a data-driven, computational approach to explore how iconicity is influenced by semantics and the modality of a language. Recent experiments have used participant ratings to capture iconicity across the lexicons of both signed languages (Caselli et al., 2017; Vinson, Cormier, Denmark, Schembri, & Vigliocco, 2008) and spoken languages (Perry, Perlman, & Lupyan, 2015; Winter et al., 2017). Here, we combine these ratings with high-dimensional distributional vectors to examine the differences, as well as similarities, in the way iconicity is distributed across the lexicons of American Sign Language (ASL) and English.

### 1.1. FACTORS THAT INFLUENCE THE ICONICITY OF SIGNS AND WORDS

The degree of iconicity of a sign or word is likely to be influenced by multiple factors (Dingemanse et al., 2015; Perlman et al., 2018; Perniss et al., 2010). Here, we examine two of these factors: the discriminability of signs and words, and the degree to which they afford iconicity, i.e., the extent to which a linguistic form can resemble its referent. A third factor, not directly examined in this paper, is the role of iconicity in the learnability of new signs and words (Imai & Kita, 2014; Laing, 2019; Perniss & Vigliocco, 2014; Perry et al., 2015).

#### 1.1.1. Discriminability

Iconicity can limit the ability to perceptually discriminate the forms of signs and words (Dingemanse et al., 2015; Gasser, 2004; Monaghan, Shillcock, Christiansen, & Kirby, 2014; Perniss & Vigliocco, 2014; Sidhu & Pexman, 2018), which may affect the dispersion of iconicity across a lexicon. In his classic work, Bühler (2011) observed that, in a language with an iconic vocabulary, the word-forms are necessarily constrained by the need to resemble their meanings. A lexicon encodes thousands of concepts spanning objects, attributes, states, events, etc., and young children must learn to rapidly distinguish these during the online flow of conversation. More concepts to encode translates to a more crowded (i.e., more dense) articulatory and conceptual space, demanding even finer-grained distinctions in form and meaning. This situation can be especially problematic because similar meanings are often used in similar contexts, which increases the likelihood of confusion. For example, the similarity between *log* and *lock* poses little confusion given the very different contexts in which these words are used. Much more confusing would be if such overlap in form existed for semantically related words like *lock* and *key*. When two lexical items are used in similar contexts, there may be strong pressure to relax the constraint of iconicity to allow their forms to vary in ways that optimize their discriminability. Thus, it is not ‘arbitrariness’ per se that confers this advantage for discriminability, but more accurately, emancipation from the iconicity constraint, i.e., the need for a form to resemble its meaning. (See Lupyan & Winter, 2018, p. 6 as an example of this common conflation.)

Gasser (2004) demonstrated the negative relationship between iconicity and discriminability with a connectionist network trained to learn a set of associations between forms and meanings (i.e., a lexicon), each represented as three-dimensional vectors in a formal or semantic space. The lexicons varied in the number of meanings they contained and whether mappings between form and meaning were arbitrary or iconic, operationalized as correlations between the semantic and formal vectors. Gasser’s model confirmed that iconic items are initially easier to learn than those with an arbitrary relationship between form and meaning. Early in the training, the network was more accurate learning the iconic items, but over the longer course of training, only small lexicons retained this advantage. In the large lexicons with more crowded formal and semantic spaces, the network became more accurate with the arbitrary lexicon, which contained more clearly discriminable items.

Evidence of a negative relationship between iconicity and discriminability has also been found in the vocabulary of a natural spoken language. Using subjective iconicity ratings of English words (Winter et al., 2017), Sidhu and Pexman (2018) tested whether words with sparser semantic neighborhoods were more iconic than those with denser neighborhoods. They found a negative relationship between iconicity and semantic neighborhood density: more iconic words were associated with sparser semantic neighborhoods. Moreover, while words with meanings high in sensory information tended to be more iconic (e.g., *buzzing*, *mushy*), this relationship held only within sparser semantic networks.

Here, we examine the idea that the relationship between iconicity and the pressure for discriminability interacts with the modality of language. Signed languages use multiple articulators such as the hands, body, and face which may offer more degrees of freedom for articulation than spoken languages. As Gasser (2004) observed, a larger form space provides greater opportunity to increase discriminability without eroding iconicity. As a result, signed languages may therefore be under less pressure to emancipate lexical forms from iconicity. Broadly in line with this hypothesis, the iconicity of signs was found to be positively correlated with phonological neighborhood density: more iconic signs tend to occur in denser phonological neighborhoods (Caselli & Pyers, 2017; Caselli et al., 2017). This suggests that the need to discriminate between sign forms does not degrade iconicity. Further support for this idea comes from the finding that more iconic signs tend to be more phonologically complex (Perniss, Lu, Morgan, & Vigliocco, 2018). This phonological complexity may permit more degrees of freedom in form, thus reducing demands on discriminability.

#### 1.1.2. Iconic affordances of the articulators

A second factor that affects the dispersion of iconicity across the lexicon of a language is the potential for aspects of the form of a signal to resemble the meaning it is used to express (Dingemanse et al., 2015; Emmorey, 2014; Perlman & Cain, 2014; Taub, 2001). The affordance of iconicity is dictated to a great extent by the language modality. For example, it is often assumed that the visual, primarily manual articulation of signed languages facilitates iconic forms that depict actions, shapes and sizes of referents, and spatial relationships, while spoken words mostly lend themselves to iconic depictions of various kinds of vocal and environmental sounds (i.e., onomatopoeia). Consequently, it is assumed that signed languages afford dramatically more iconicity than the limited amount possible in spoken languages (Armstrong & Wilcox, 2007; Hockett, 1978; Sandler, 2013). Casual observation of signed languages suggests that their vocabularies are highly iconic, with some subjective analyses finding that over a half or more of signs contain elements with identifiable links between their form and meaning (Pietrandrea, 2002; Wescott, 1971). Such a conclusion is also supported by experiments testing the ability of participants to generate novel signals for various meanings in gesture compared to vocalization (Fay, Lister, Ellison, & Goldin-Meadow, 2014; but see Perlman & Lupyan, 2018).

However, the amount of sign iconicity may vary depending on how iconicity is operationalized, and there is some debate about how iconicity can best be objectively measured (see Motamedi, Little, Nielsen, & Sulik, 2019, for discussion). The ability to identify an iconic relationship between form and meaning depends on one’s linguistic and cultural experience (e.g., Occhino, Anible, Wilkinson, & Morford, 2017), and the perception of iconicity might be mediated by experience with linguistic patterns within the lexicon itself (Occhino, Anible, & Morford, 2020). In fact, the lexicon of signed languages may be overall less iconic than previously thought. Subjective iconicity ratings of 993 ASL signs indicated that signs were heavily skewed toward the less iconic end of the scale, regardless of whether the ratings came from deaf signers or hearing sign-naive participants (Caselli et al., 2017; Sevcikova Sehyr, & Emmorey, 2019). Additionally, the meanings of most signs are not immediately obvious from their form – only the meanings of a small proportion of signs are guessed correctly by sign-naive participants (e.g., Bellugi & Klima, 1976; Sevcikova Sehyr & Emmorey, 2019). Although it seems likely that a greater proportion of sign than word meanings might be correctly guessed by naive participants and that signs might be rated as more iconic than words, it nonetheless remains to be determined empirically whether signed languages are, in any sort of absolute terms, actually more iconic than spoken languages.

At the same time, considerable research now shows that iconicity is widespread across the vocabularies of spoken languages, extending far beyond the semantic domain of sound. Cross-linguistic studies of ideophones shows that this nearly universal word class of depictive words is used to communicate about a rich array of meanings related to sensory, motor, and cognitive experience (Dingemanse, 2012). Studies of English and Spanish, standard European languages that are often claimed to be scarce in ideophones (Perniss & Vigliocco, 2014), find that iconicity can be prevalent in different word classes too, including verbs and adjectives (Perry et al., 2015), and generally in words with sensory-related meanings, particularly those related to touch in addition to sound (Winter et al., 2017).

A recent approach to the systematic study of iconicity across the lexicons of different languages is to interrogate iconicity ratings in which participants assess the degree of resemblance between the form of a sign or word and its meaning. This method operationalizes iconicity as a substance that comes in degrees (Dingemanse, Perlman, & Perniss, 2020), and in doing so, abstracts away from the particular type of iconic mapping at play. Such aggregation of ratings obscures variability in individuals’ subjective assessments of form–meaning pairings, but the advantage of this approach is that it enables analysis of hundreds or thousands of items, allowing systematic study of large swaths of a lexicon (see Winter, 2019). Mean ratings for items can be compared between different portions of a vocabulary that vary in characteristics of interest, such as lexical class, phonological properties, age of acquisition, sensory characteristics, and frequency.

A related advantage of iconicity ratings is that – when collected in commensurate ways – they can serve for comparative analyses of iconicity between different languages. In one recent study, previously collected iconicity ratings were used to compare the semantic patterns of iconicity in two historically unrelated signed languages, British Sign Language (BSL) and ASL, with two spoken languages, English and Spanish (Perlman et al., 2018). The ratings were obtained by asking participants to indicate the degree to which a spoken word or sign resembles its meaning. Iconicity of signs, but not words, was positively correlated with participant ratings of concreteness (based on English words, as were the following semantic ratings), whereas all four languages were, to some degree, associated with ratings of imageability and sensory experience. In both signed languages, as well as English, there was a positive relationship between iconicity and the level of haptic experience associated with the meaning of the lexical item. The iconicity of words – but unsurprisingly, not signs – was positively related with auditory experience. Contrary to Perlman et al.’s expectations, the iconicity of signs turned out to be negatively correlated with the level of visual experience associated with the meaning. The authors speculated that this could be a consequence of the highly detailed nature of visual imagery, which could only be reconstructed into a schematic representation in manual signs.

Perlman et al. (2018) also conducted a narrower semantic analysis of just the 220 meanings for which there were iconicity ratings in all four languages. In ASL and BSL, iconicity was rated highest for the meanings categorized as ‘verbs’ according to their English translations. Verbs were rated high in iconicity in English too, but not in Spanish (also see Perry et al., 2015). Notably, Spanish, unlike English, is a satellite-framed language that typically expresses manner of motion information with adverbials, instead of with the verb (Talmy, 1985). Those meanings labeled as ‘adjectives’ in English were rated high in iconicity in both spoken languages, but not in the signed languages. More fine-grained semantic analysis found that, for signs, manual actions and body parts were rated as most iconic of all, while colors were rated lowest in iconicity. For words, iconicity was relatively high in meanings related to various properties (e.g., *quiet*, *slow*, *wet*), and English – but not Spanish – patterned like ASL and BSL with respect to high iconicity in meanings related to locomotion and manual actions. However, compared to the signed languages, the spoken languages did not show such clearly delineated domains in which iconicity was elevated (although, notably, sound-related words were not part of this subset).

Considered altogether, research using iconicity ratings – although lacking rich detail about the particular iconic mappings involved – is nevertheless helping to uncover the specific ways that iconicity is distributed across the vocabularies of different languages. The findings show that this distribution is not haphazard, but rather, certain kinds of words and signs tend to be iconic because iconicity serves important cognitive and communicative functions. Indeed, by considering factors such as discriminability and the iconic affordances of the vocal versus manual articulators, we can make specific predictions about which words and signs ought to be iconic, and, by and large, these predictions tend to bear out.

### 1.2. CURRENT STUDY

In the current study, we extend the use of iconicity ratings by taking a computational, data-driven approach to examine how iconicity is distributed across the vocabularies of a spoken language (English) compared to a signed language (ASL). We show that modality-specific affordances for iconicity can be automatically identified using a high-dimensional, distributional representation of lexical semantics. Our analysis asks how accurately we can predict previously collected iconicity ratings in English and ASL using a distributional model of lexical semantics that has been constructed from large-scale, naturally occurring English data. We find that both sets of iconicity ratings can be systematically projected onto this representation of meaning, but that these projections differ in intriguing ways. This result demonstrates that it is possible to identify semantic correlates of iconicity in a bottom-up, data-driven manner, and that the procedure we introduce is sensitive enough to identify language- or modality-specific patterns.

We then use this model of lexical semantics to examine the relationship between iconicity and semantic density in the two languages. Previous research has demonstrated a negative relationship between iconicity and semantic density in English: meanings with close semantic neighbors are less likely to be expressed using iconic word-forms (Sidhu & Pexman, 2018). But it is currently unclear whether this relationship also characterizes the distribution of iconicity in a signed language. We find that, on the scale of the vocabulary at large, the same negative relationship between iconicity and semantic density holds in ASL as in English. However, among highly iconic items, this relationship is inverted in ASL, which contains pockets of highly similar, but highly iconic signs. We consider the limitations of using a model of English semantics for the ASL analysis in the ‘Discussion’.

## 2. Methods

The complete analyses and code can be found in the Open Science Framework Repository <https://osf.io/znbcu/>.

### 2.1. ICONICITY RATINGS

We compiled previously collected experimental ratings of iconicity in English and American Sign Language: 3000 ratings were available in English, and 961 ratings were available in ASL, with 552 meanings with ratings in both languages (i.e., the meaning intersection). English ratings were collected on a −5 to 5 scale (see detailed instructions in Perry et al., 2015), with −5 indicating “words that sound like the opposite of what they mean”, 0 “words that do not sound like what they mean or the opposite”, and 5 “words that sound like what they mean”. The raters were all native speakers of English. ASL ratings were on a 7-point scale indicating how much a sign “looks like what it means”, with 1 indicating signs that are “not iconic at all” and 7 those that are “highly iconic” (Caselli et al., 2017). Signs were rated by hearing sign-naive participants who were given the English translation of the ASL sign. These ratings were placed on a comparable scale by subtracting the mean of the ASL ratings from all ASL ratings and subtracting the mean of the English ratings from the English ratings.

### 2.2. DISTRIBUTIONAL MODELS

We obtained a model of English lexical semantics which characterizes word meaning as vector embeddings in a high-dimensional semantic space, resulting in word vectors. The word vectors we used in this analysis were generated by Facebook Artificial Intelligence Research (Bojanowski, Grave, Joulin, & Mikolov, 2017). These vectors were trained by applying the Skipgram algorithm to a large volume of naturally occurring English text drawn from Wikipedia. The algorithm uses lexical and sublexical co-occurrence statistics to induce 300-dimensional semantic vectors for all words in the Wikipedia corpus. The result was that words which were similar in meaning had similar vector representations. This model, like others trained using similar methods, has been shown to capture a wide range of semantic and relational information (Chen, Peterson, & Griffiths, 2017; Hollis & Westbury, 2016; Nematzadeh, Meylan, & Griffiths, 2017; B. Thompson & Lupyan, 2018). We were able to obtain vectors for words corresponding to 2964 of the 3000 English words that had been rated for iconicity and to 923 of the 961 rated ASL signs, and for all 552 meanings with ratings in both languages. Details on the sets of English words and ASL signs used in the analyses can be found in the OSF Repository.

### 2.3. PREDICTING RATINGS FROM VECTORS

One way to assess whether a distributional model of lexical semantics contains information relevant to the patterning of iconicity through a lexicon is to ask: How accurately can the (previously collected) iconicity ratings be predicted from the model? To find out, we predict the (standardized) iconicity rating of a meaning from its vector representation using a regression analysis (more specifically, ridge regression). The prediction accuracy should reflect the extent to which vector representations contain information that is relevant to the semantic factors that afford iconicity. For example, word vectors have been shown to implicitly capture factors such as lexical concreteness, word frequency, and valence, among others (Hollis & Westbury, 2016). If the factors being captured by word vectors are relevant to iconicity, we should be able to capture variance in iconicity ratings by using these vectors as semantic predictors. Similar techniques have been applied to the analysis of other lexical features, such as valence, arousal, and dominance (Hollis, Westbury, & Lefsrud, 2017; B. Thompson & Lupyan, 2018).

### 2.4. COMPUTING SEMANTIC DENSITY

We computed three measures of semantic density for English. All measures relate to the distance between meanings in semantic space as determined by their word vectors. In line with common practice, we used cosine similarity in vector space as a measure of semantic similarity (words with small cosine distance, or larger cosine similarity, between their word vectors are semantically similar). All measures were computed with respect to the 50,000 most frequent English words. Global Centrality is the sum of a word’s cosine similarity with all 50,000 other words. This measure captures the overall connectedness of a word through the entire vocabulary, and is therefore likely to be high for words that occur in many contexts, such as function words. Neighbor concentration is the sum of the cosine similarities between a target word and its *k* closest semantic neighbors. In contrast to global centrality, this measure is intended to capture density in high-similarity structure. A word can have high neighbor concentration either by having a small number of highly semantically similar neighbors, or a larger number of moderately semantically related neighbors. In our analyses *k* varied between 5 and 100. Finally, we defined local concentration, which is the sum of a word’s cosine similarity to all other words which exceed a similarity threshold (defined somewhat arbitrarily as the mean of the overall distribution of cosine similarities among the union of words rated in both languages, plus one standard deviation in this distribution). This measure relaxes the restriction that *k* (the number of words considered semantic neighbors) is fixed across words. Table 1 provides some examples of words with particularly high and low density according to these measures.

## 3. Results

### 3.1. PREDICTING ICONICITY FROM WORD VECTORS IN ENGLISH & ASL

**Fig. 1.**
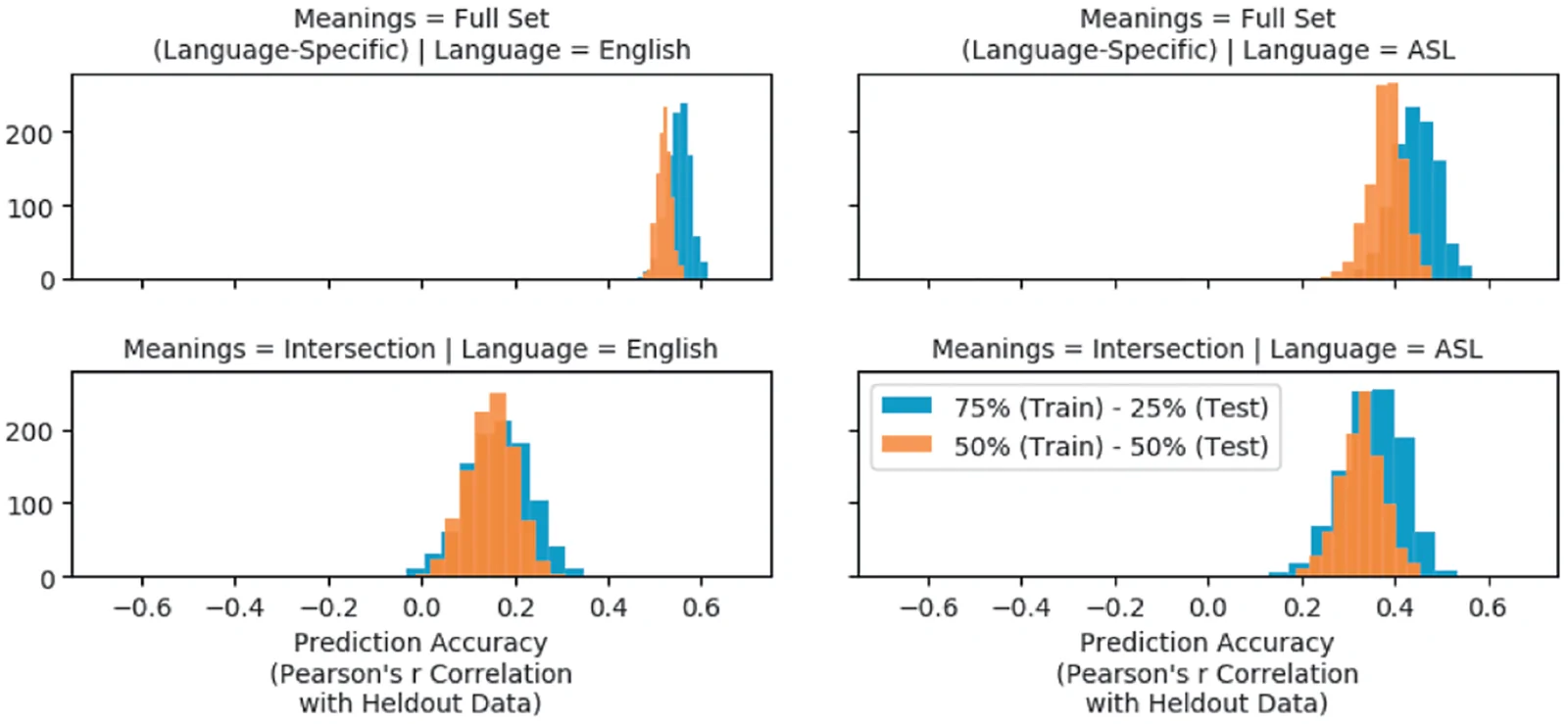
Distribution of accuracy scores when predicting iconicity from word vectors.

Figure 1 shows that we were able to predict iconicity ratings from word vectors in both English and ASL. The figure shows the distribution of accuracy scores over one thousand replications of the prediction procedure outlined in section 2.3. Prediction accuracy is given by the Pearson correlation coefficient describing the relationship between predicted iconicity ratings and the true iconicity ratings (among a held-out set of test meanings which were not used to infer the regression coefficients). The top row shows the distribution of accuracies when predicting iconicity ratings from 1000 randomly sampled train-test splits (blue bars show accuracy scores based on a 75–25% train-test split; orange bars show results of a more conservative 50–50% train-test split) of the full set of meanings (English translations) for which iconicity ratings and vector representations are available in ASL (left, 887 total ratings) and English (right, 2964 total ratings).

Not surprisingly, prediction is more accurate in English than in ASL for the full training sets. There are two procedural reasons to expect this. First, the semantic model was induced from English text, and second, there are more iconicity ratings in English, and therefore more data from which to infer a model. Nonetheless, it is instructive that prediction of ASL iconicity ratings from a distributional model of lexical semantics is possible, even if that model is based on English text. The model’s predictive success suggests that lexical semantic density may not be wildly different across ASL and English, although this awaits confirmation based on semantic density data from ASL. The bottom row of Figure 1 shows the distribution of accuracy scores when performing the same procedure on only meanings that have been rated for iconicity in both ASL and English (the meaning intersection). This case is informative because it corrects for the data imbalance mentioned above. This smaller set of data (277 total meanings) leads to weaker predictions in both languages, but, in this case, the prediction is actually stronger for ASL. Together these results suggest that the distributional model of lexical semantics we have analyzed contains information relevant to the semantic distribution of iconicity in both English and ASL.

### 3.2. VISUALIZING ICONICITY IN SEMANTIC SPACE

**Fig. 2.**
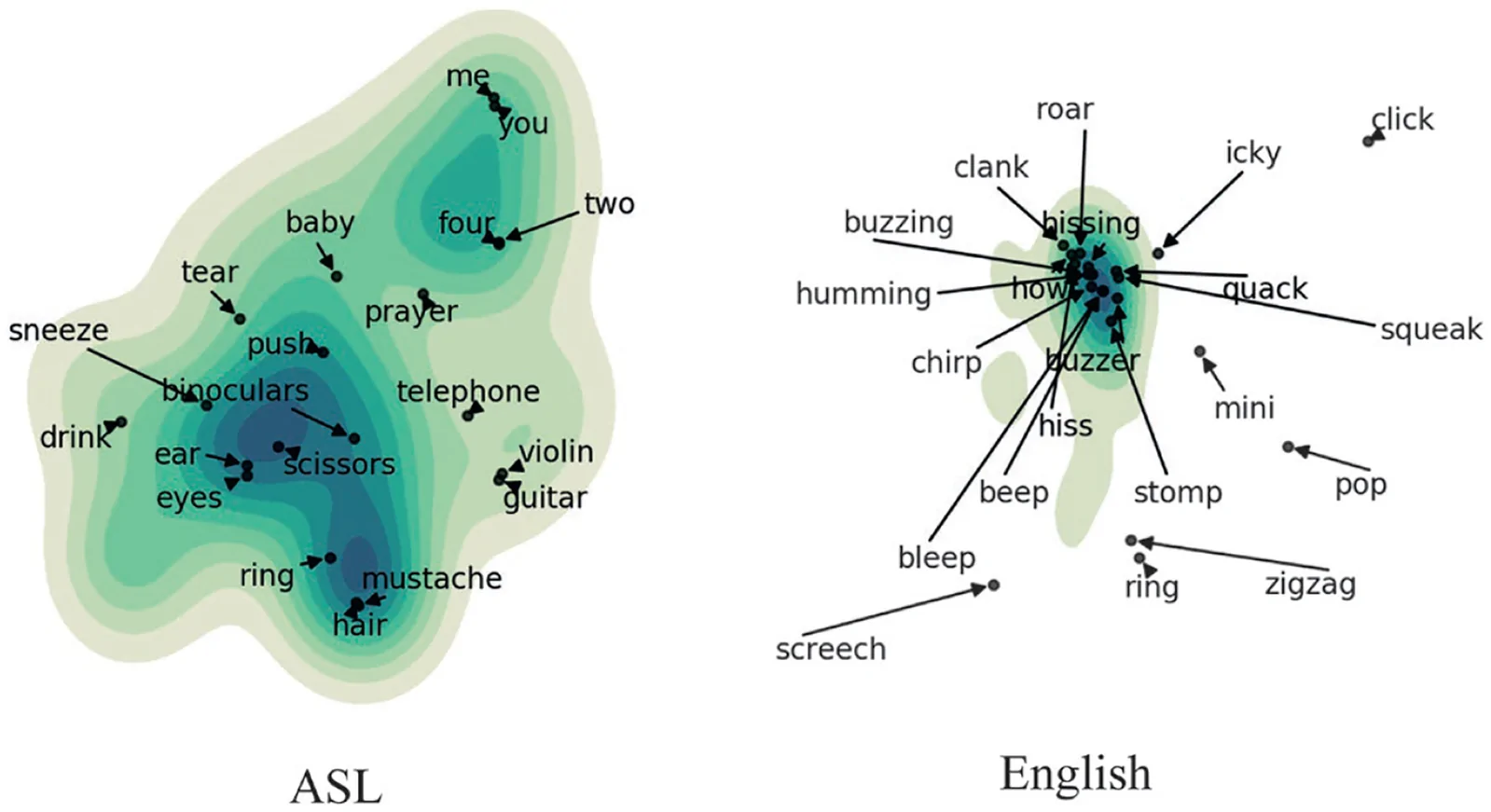
Meanings rated high in iconicity projected onto a two-dimensional semantic space. The top 20 most iconic forms in each language are labeled. Darker colors represent regions of semantic space with higher iconicity.

Figure 2 shows the semantic distribution of highly iconic forms (standardized iconicity rating larger than 1.5) in ASL and English projected onto a two-dimensional semantic space. Even in this highly simplified space, language-specific distributional patterns are clear. To create Figure 2, we obtained word vectors for 3409 meanings which have been rated for iconicity in either ASL or English (the union of their meanings). We then performed dimensionality reduction on these vectors in two steps: first using PCA to estimate 50 principal components of the embedding space implied by these vectors, then applying the T-SNE (t-distributed stochastic neighbor embedding) algorithm (Maaten & Hinton, 2008) using a cosine similarity metric to the induced component loadings. This resulted in a two-dimensional embedding of the union meanings in a shared semantic space. For both languages, we identified meanings that were rated as iconic, and used these to infer a language-specific kernel density estimate over the two-dimensional embedding space.

**Fig. 3.**
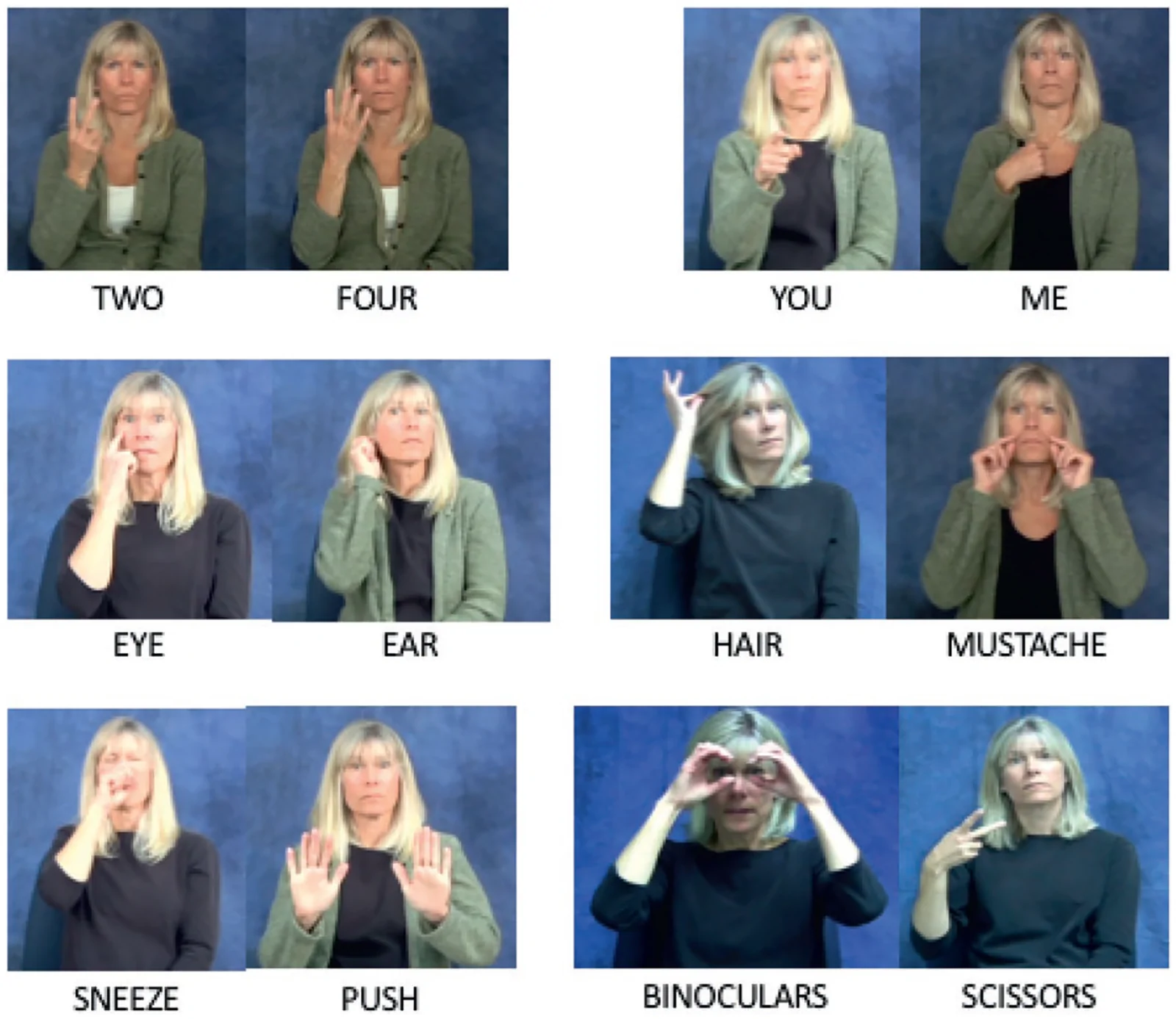
Illustrations of ASL signs from semantic clusters found to be high in iconicity.

Most notably, iconic meanings are spread more widely over semantic space in ASL, which indicates more distinct and more dispersed clusters of iconicity than English (see Figure 2). For example, in ASL, distinct clusters that relate to number (e.g., TWO, FOUR), personal pronouns (YOU, ME), parts of the body (EYE, EAR), and the head (HAIR, MOUSTACHE) are visible (see Figure 3 for sign illustrations). In contrast, iconic meanings in English are distributed around a single tight cluster of meanings related to sound (e.g., *hiss*, *quack*, *squeak*). There are highly iconic meanings in this same region of semantic space (relating to sound) in ASL, but many fewer (and these items refer to the source of the sound). Figure 2 reinforces the analysis reported in section 3.1 by demonstrating that the semantic model contains information relevant to the patterning of iconicity.

### 3.3. semantic density and iconicity

**Fig. 4.**
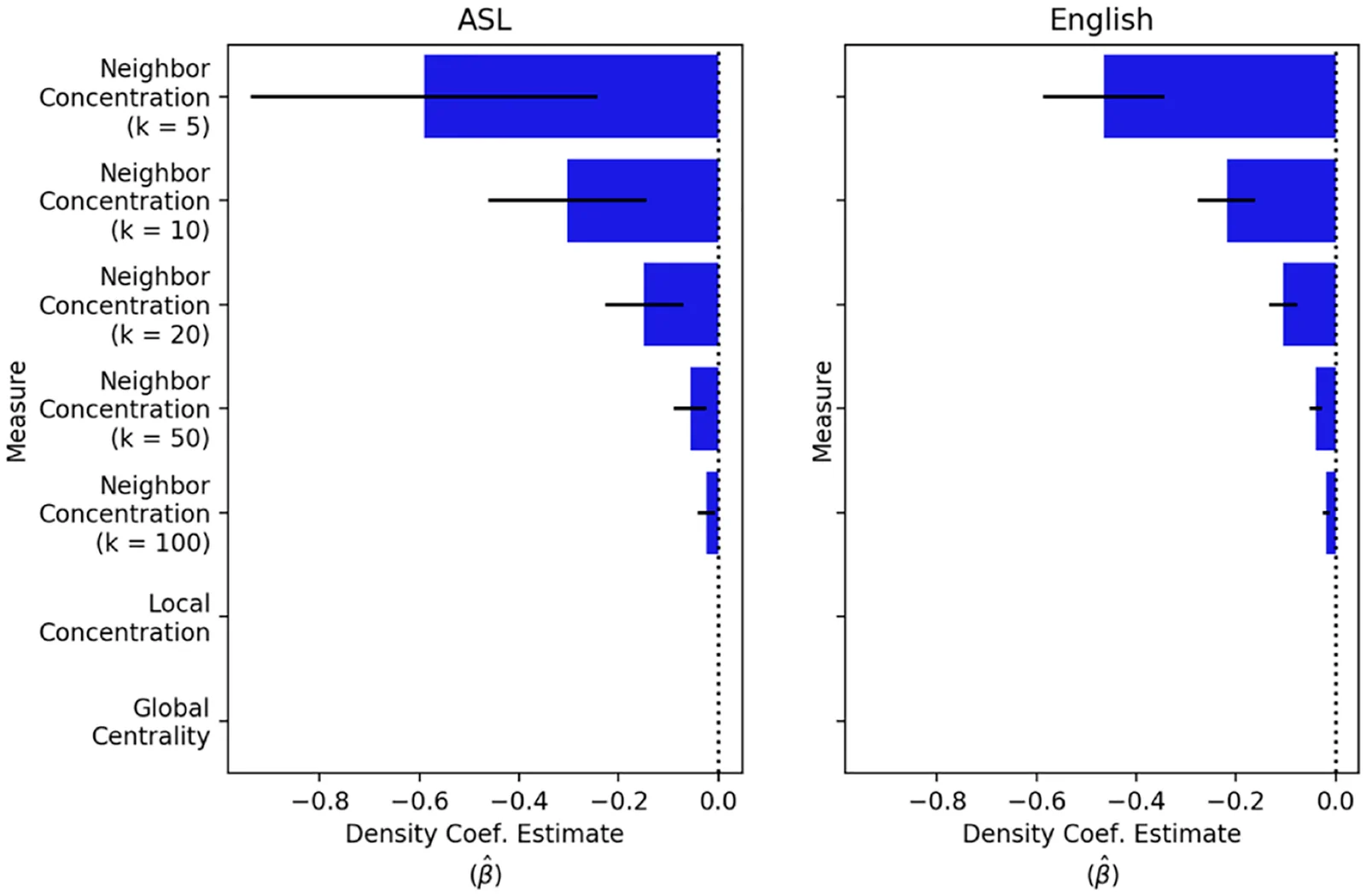
Maximum likelihood coefficient estimates in regression analyses predicting iconicity from semantic density (controlling for frequency). Bars show standardized coefficient estimates. Black lines show 95% confidence intervals.

Using the distributional model of word meaning and our new measures of semantic density, we replicated Sidhu and Pexman’s (2018) finding that semantic density is negatively related to iconicity ratings of English words. We examined the relationship between density and iconicity in English and ASL by performing a separate ordinary least squares regression analysis to predict standardized iconicity from language-specific word frequency and each semantic density measure. Figure 4 shows estimates of the regression coefficient for semantic density in all cases where we observed a significant effect (at *p* < .05). We find the same negative relationship between semantic density and iconicity ratings in ASL that has been observed in English. If anything, this pattern appears more pronounced in ASL than in English, although more iconicity ratings in ASL would be required to be confident in this distinction between languages.

**Fig. 5.**
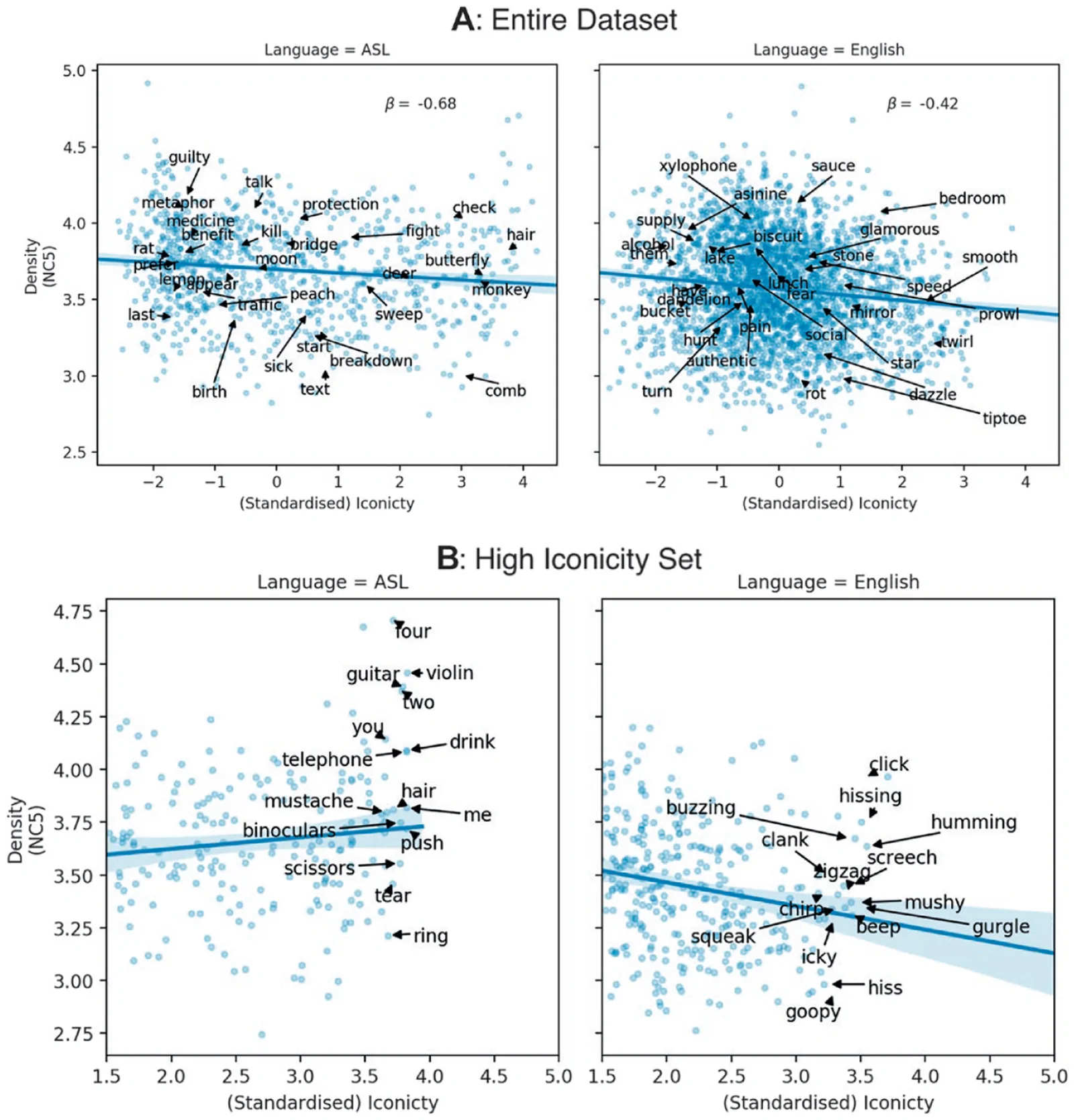
Relationships between semantic density and iconicity, controlling for frequency in ASL (left) and English (right) for the entire dataset (top) and focusing only on high-iconicity signs and words (bottom).

Figure 5 shows the relationships between semantic density (Neighbor Concentration; *k* = 5), iconicity, controlling for English frequency (Balota et al., 2007) and ASL subjective frequency (from Caselli et al., 2017). The relationship between iconicity and semantic density in both languages appears to be strongest when density is measured in terms of a word’s closest semantic neighbors (lower *k*). Overall, the same pattern of relationships holds between iconicity and semantic density in both ASL and English. However, when we look specifically at meanings with highly iconic forms, we find an interesting difference between languages, particularly among nouns. The bottom panels in Figure 5 show the relationship between iconicity and semantic density among the meanings rated as most highly iconic in both languages. English displays a strong preference against the most iconic words being in dense semantic neighbors (no words in the top right of the plots). However, ASL does not show this same tendency. In fact, among highly iconic items, the relationship between iconicity and density is inverted: the most iconic signs in ASL occupy semantically dense neighborhoods.

## 4. Discussion

Our study builds on recent work documenting the systematic patterns of iconicity across different kinds of lexicons, including signed and spoken languages (Dingemanse et al., 2015; Padden et al., 2015; Padden et al., 2013; Perlman et al., 2018; Perniss et al., 2010). We took a data-driven, computational approach to examining iconicity in the vocabularies of ASL and English. Using a high-dimensional distributional model of English lexical semantics, we analyzed how the rated iconicity of signs and words is concentrated across different domains of meaning, as well as its relationship with semantic density. The first outcome of our analyses is that models of spoken language lexical semantics drawn from large corpora of English text proved to be useful for predicting the iconicity of ASL signs, as well as English words. The models were able to predict iconicity ratings in both languages with a moderate degree of accuracy. In fact, when the training sets were equivalent, the model was better at predicting the iconicity of ASL vocabulary than English. This result may indicate a more systematic distribution of iconicity in ASL, perhaps reflecting a greater level of iconicity overall.

Second, when we visualize the distribution of iconicity of each language in two-dimensional semantic space, we found that there appear to be more distinct pockets of iconicity in ASL than in English (Figure 2). ASL shows four clear concentrations of iconicity, whereas English has just one. A look at the most iconic meanings in this dataset shows that iconicity in ASL signs is often highest in those related to bodily actions (e.g., SNEEZE, PUSH), body parts (EYES, HAIR), manipulable objects (BINOCULARS, SCISSORS), personal pronouns (YOU, ME), and numbers (TWO, FOUR) (see Figure 3). In contrast, iconicity in English is concentrated most distinctively in sound-related words. This finding indicates that the distribution of iconicity across ASL signs is more widely systematic than across English words. While it is not direct evidence that ASL is *more* iconic than English, it is consistent with this claim, and fits with previous research finding that iconicity is more correlated between ASL and BSL signs with the same meaning compared to English and Spanish words (Perlman et al., 2018).

Third, we found that iconicity decreases in response to pressures on discriminability, both in English – reproducing previous results (Sidhu & Pexman, 2018) – and notably in ASL as well. In both languages, there was an overall negative relationship between semantic density and the iconicity of words and signs, which was strongest for the closest semantic neighbors (*k* = 5). This negative relationship might be predicted for the global centrality measure where items with more abstract grammatical functions (e.g., *but*, *and*, *that*) have the most neighbors, but do not easily afford iconic depiction. However, the negative relation held even with broader definitions of what counts as a neighbor (Figure 4). This pattern fits with what would be expected if discriminability was driving the effect, being hardest among closest neighbors. Thus, at the overall vocabulary level, there may be a universal pressure for languages to evolve less iconic forms in order to reduce semantic confusion among neighbors.

Interestingly, though, when we zoom in on the most iconic lexical items in each language – those 1.5 standard deviations or more from the mean – we find a reversal of this relationship in ASL, but not in English. That is, in ASL, the most iconic signs are actually relatively semantically dense, suggesting that, for these meanings, the drive for discriminability may be overridden by other factors. One factor that may reduce discriminability demands is that a number of these highly iconic signs have meanings that involve bodies (ME, YOU), body parts (HAIR, MOUSTACHE), and bodily actions relating to the manipulation or handling of objects (PUSH, BINOCULARS). Closely related meanings in these domains may be more readily discernible by perceivers because of their extensive, very direct experience with real bodies and bodily actions. Our ability to visually discriminate among human body parts and body actions may permit highly iconic signs to inhabit these dense semantic neighborhoods. In several instances, the incorporation of indexical elements (i.e., pointing) may help to distinguish the salient body part. Another factor, not examined here, is that highly iconic signs may be more phonologically complex, allowing more degrees of freedom which increases discriminability among forms (Perniss et al., 2018).

Previous analyses with iconicity ratings have focused on intuitive semantic categories like ‘sound’ or ‘manual actions’. Such categories are useful abstractions, but they can underspecify the semantic representations exploited by users of natural languages. The word *roar*, for example, is clearly related to sound and is highly iconic in spoken English. But it is also related to animals, and perhaps to the emotion of anger; in addition, it is rated as highly concrete by speakers of English, and occupies a relatively sparse semantic neighborhood. The approach used here has allowed us to identify semantic groupings from the bottom up, which helps to determine whether researcher intuitions regarding the semantic categories of interest are indeed the ones that are operating. This approach may also serve to discover new phenomena and motivate new hypotheses. For example, here the finding that highly iconic signs in ASL tend to be more, not less, semantically dense has generated the hypothesis that these pockets might be comprised of signs representing body parts and bodily actions, with which we have extensive experience discriminating in the real world.

We also note that, along with the innovations in our methodological approach, there are some important limitations to consider. For one, our analysis is based on a model of semantics inferred from English text, rather than from ASL discourse. It is not yet clear how these vectors generalize to the semantics of other languages, especially a signed language like ASL. Prior research indicates that (spoken) languages that are more closely related tend to have more similar meaning spaces as captured by distributional semantics (B. Thompson, Roberts, & Lupyan, 2018), and research also shows that ratings of lexical concreteness in Dutch can be predicted using a technique that relies on English distributional semantics and vice versa (B. Thompson & Lupyan, 2018). However, while these results are reassuring, they are limited to two closely related spoken languages. A related issue concerns the specific interpretation of our semantic density measures, particularly as we manipulated the values of *k*, the number of neighbors. Future research should investigate different approaches to semantic density and their relationship to iconicity more comprehensively.

A second limitation of our approach is that the sampling of English words and ASL signs for which we have iconicity ratings was determined opportunistically from previous studies. Consequently, the overlap with distributional vectors is partial and not a strategic representation of the vocabularies of either language. Notably, the iconicity ratings for signs used here, and in previous studies (Caselli et al., 2017; Vinson et al., 2008), include a relatively narrow subset of the lexicon, lacking, for example, fingerspelled words and productive classifier/depicting signs. Thus, there is a need for iconicity ratings that span larger sets of words and signs, including more representative coverage across different semantic domains selected for the purpose of making comparisons between languages. Furthermore, it is critical that iconicity ratings to be compared between different languages are collected according to instructions and scales that are as commensurate as possible. In the current work, noise may have been introduced as a result of the different scales used: −5 (opposite) to 0 (arbitrary) to 5 (iconic) scale for English and 1 (arbitrary) to 7 (iconic) for ASL.

Finally, a third limitation is that the iconicity ratings of ASL signs were elicited from non-signers of ASL based on English translations of these signs. Past work has found that deaf signers rate the iconicity of signs of their own language somewhat differently from signers of other languages (Occhino et al., 2017) and from hearing non-signers (Sevcikova Sehyr & Emmorey, 2019), even though signers’ and non-signers’ iconicity ratings were highly correlated (*r* = 0.82).

In light of these limitations, our study serves as an exploratory investigation, particularly with respect to ASL. In future work, it is especially important to move beyond English – not just as the language of investigation, but also in the use of English-speaking raters and the use of semantic representations derived from English texts. Similar data-driven studies of iconicity across diverse languages, with diverse raters and diverse texts, are key to assessing the generalizability of the kinds of iconic patterns found here.

## 5. Conclusion

Considered together, the current findings contribute to an increasingly detailed picture of how iconicity is distributed across different languages. They add to an expanding range of studies which show that iconicity is patterned in predictable ways across vocabularies, resulting in part from its role in the psychological processes involved in using language. Depending on the particular characteristics of a given language – such as whether it is spoken or signed – these processes can drive selective pressures on different portions of vocabulary. Here we found further evidence that the potential for iconicity to interfere with the discriminability of signs and words may drive the erosion of iconicity over time, although this effect may be reduced in certain semantic domains of vocabulary within signed languages. We also found support for the widely assumed premise that signed languages afford, if not more iconicity per se, more systematic, readily detectable patterns of iconicity across their vocabulary than spoken languages. In addition to the factors examined here, the level of iconicity may also be influenced by its role in learning processes, helping both children and adults to learn new signs and words (Imai & Kita, 2014; Laing, 2019; Ortega, 2017; Perniss & Vigliocco, 2014; Perry, Perlman, Winter, Massaro, & Lupyan, 2018). For example, the early vocabularies of children show a preponderance of iconic items, a pattern found in English and Spanish (Perry et al., 2015) as well as in British Sign Language (R. Thompson, Vinson, Woll, & Vigliocco, 2012) and ASL (Caselli & Pyers, 2017).

Thus, there are at least three important factors that influence iconicity across the vocabulary of a language. These factors may operate somewhat differently in different language modalities, but they nevertheless appear common to all languages. However, much more research is needed to understand the complex patterns of iconicity across languages, especially work with a far more diverse array of signed and spoken languages. This research will surely be complemented by laboratory work with artificial symbol systems (Garrod, Fay, Lee, Oberlander, & MacLeod, 2007; Little, Eryilmaz, & Boer, 2017; Perlman, Dale, & Lupyan, 2015; Verhoef, Kirby, & Boer, 2016). Ultimately, we may aim to develop models that account for the level of iconicity across the vocabulary of diverse languages, including how this iconicity can be expected to change over historical time.

## Figures and Tables

**TABLE 1.** Some words with high and low semantic density according to three distinct measures

	High	Low
Neighbor Concentration (*k* = 5)	*February, four, saxophone*	*boomerang, grr, open*
Local Concentration	*honest, giggling, whatever*	*full, mat, under*
Global Centrality	*but, that, things*	*file, list, title,*
